# Mathematical modeling and parametrical analysis of the temperature dependency of control drug release from biodegradable nanoparticles

**DOI:** 10.1039/c9ra00821g

**Published:** 2019-03-15

**Authors:** Armando Lucero-Acuña, Cindy Alejandra Gutiérrez-Valenzuela, Reynaldo Esquivel, Roberto Guzmán-Zamudio

**Affiliations:** Department of Chemical and Environmental Engineering, University of Arizona Tucson USA guzmanr@email.arizona.edu +1 520 6216048 +1 520 6216041; Department of Chemical and Metallurgical Engineering, University of Sonora Hermosillo MEXICO; Nanotechnology Graduate Program, Department of Physics, University of Sonora Hermosillo MEXICO

## Abstract

In this study we describe a mathematical analysis that considers the temperature effects of the controlled drug release process from biodegradable poly-d,l-lactide-*co*-glycolide (PLGA) nanoparticles. Temperature effects are incorporated and applied to two drug release models. The first one consists of a two-stage release process that considers only simultaneous contributions of initial burst and nanoparticle degradation–relaxation (BR model). The second one is a three release stage model that considers, additionally, a simultaneous drug diffusion (BRD model) step. In these models, the temperature dependency of the release parameters, initial burst constant, *k*_b_, the rate of degradation–relaxation constant, *k*_r_, time to achieve 50% of release, *t*_max_, and effective diffusion coefficient constant (*D*_e_), are determined using mathematical expressions analogous to the Arrhenius equation. The temperature dependent models are used to analyze the release of previously encapsulated Rhodamine 6G dye as a model drug in polyethylene glycol modified PLGA nanoparticles. The experimental data used to develop the mathematical model was obtained from release studies carried out in phosphate buffer pH 7.4 at 37 °C, 47 °C, and 57 °C. Multiphasic release behaviors with an overall increase rate associated with the incubation temperature were observed. The study incorporates a parametrical analysis that can evaluate diverse temperature variation effects of the controlled release parameters for the two models.

## Abstract 2

Temperature dependence of the main parameters involved in the drug release process from biodegradable nanoparticles.

## Graphical abstract

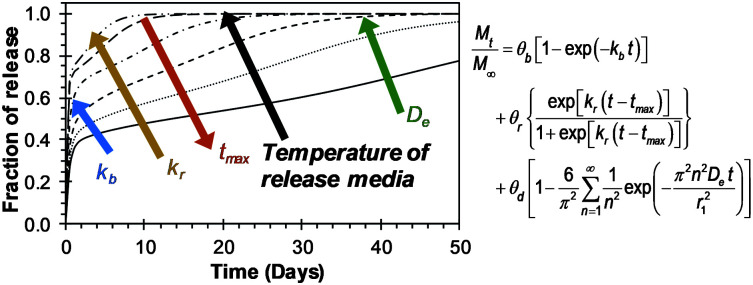

## 1 Introduction

Different biodegradable polymers that respond to external signals, like temperature, pH, light ultrasound, *etc.* have been extensively studied in the preparation of nanoparticles for drug delivery.^1–3^ These nanoparticles could be tuned to offer several benefits over free drugs; for example, they could protect the drug from undesired interactions with other organic tissues, as well as target specific tissues, boosting desired interactions with the target, and could help to control drug release. Nanostructures prepared with poly-d,l-lactide-*co*-glycolide (PLGA) are capable of controlling the drug release process by controlling several factors and by responding to some external factors. Factors, such as temperature, pH, nanoparticle size, polymer molecular weight, polymer composition, nanoparticle processing, drug hydrophobicity, drug loading, and interactions like drug–drug and polymer–drug, could influence the release of drugs from PLGA nanoparticles.^4–6^ However, among these factors, temperature plays one of the most relevant roles in the drug release process from PLGA micro and nanoparticles. For example, degradation rates of PLGA microspheres were found to increase with increasing incubation temperature.^7^ The glass transition temperatures (*T*_g_) of PLGA copolymers are above the physiological temperature of 37 °C, and hence they are glassy in nature.^8^ However, the effect of temperature over drug release could be extended to other polymers as well. Experimental release of doxorubicin at different temperatures from poly(*N*-isopropylacrylamide-acrylamide-allylamine)-coated magnetic nanoparticles shows that temperature plays a significant role and a significant release rate increase was observed with an increase in temperature.^9^ The understanding of the properties of controlled drug release rates from biodegradable systems could translate into developments to improve therapeutic techniques. Mathematical modeling of drug release could help to describe better and understand the properties and mechanisms involved in drug release. These mechanisms of drug release could include an initial burst phase, nanoparticle degradation–relaxation phase, and a fickian diffusion phase. Several mathematical models have been proposed to describe the mechanisms of drug release,^10–14^ but generally, in most of these models, temperature is not incorporated. The need for mathematical models that include diverse physical factors is evident. For instance, the incorporation of temperature in mathematical models for drug release might help elucidate and explain more effectively drug release phenomena. One approach to including temperature dependency over the degradation of microparticles of PLGA was reported in the literature by relating the degradation rate of microparticles to temperature, by using a mathematical expression of the form of the Arrhenius equation.^7^ In general, when biodegradable systems are analyzed, usually a multiphasic release behavior, especially for hydrophobic drugs, is observed.^15–18^ Models that consider more than one mechanism of release to explain this multiphasic release behavior have been used; however, the temperature effects in these models are not usually contemplated. The mechanism of initial burst combined with the bulk degradation of the polymer has been used to describe biphasic profiles of release in micro and nanoparticles.^19,20^ In these works, initial burst was analyzed using a first order equation, and the bulk degradation of the polymer was described using an analogous form of Prout–Tompkins equation.^21^ Similarly, an initial burst first-order mechanism was combined with fickian diffusion to describe drug release in polymeric microspheres.^22–24^ Likewise, multiphasic release behavior from biodegradable polymeric microparticles has been explained using triphasic models that consider mechanisms of diffusion, drug dissolution, and polymer erosion.^25^ Multiphasic drug release from as the time-sequential combination of three mechanisms of release films has been described: first-order burst release, first-order bulk degradation of the polymer, and fickian diffusion release.^26^ Also, a triphasic model that describes the overall release of hydrophobic drugs from PLGA nanoparticles by a simultaneous combination of first-order burst release, Prout–Tompkins analogous nanoparticle degradation–relaxation, and diffusional release have been described in the literature.^27^

In this work, the release of Rhodamine 6G (R6G) from PEGylated PLGA nanoparticles (R6G-PNP-PEG) was determined at three different temperatures. The data was analyzed mathematically by considering two different multi-stage release models that incorporate temperature effects over the entire drug release process. The two models consider simultaneous contributions of two (BR model) or three (BRD model) mechanisms of drug release. The mechanisms in the first model (BR model) involve a first-order burst release and the controlled release by the bulk degradation of the polymer. The second model (BRD model), incorporates a fickian diffusion release stage additionally. The temperature dependency of the release parameters, initial burst constant, (*k*_b_), rate of degradation–relaxation constant, (*k*_r_), time to achieve 50% of release, (*t*_max_), and effective diffusion coefficient constant (*D*_e_), were determined using mathematical expressions analogous to the Arrhenius equation. A parametric study for each one of the two models was performed to better understand the effects of temperature variations over the controlled release parameters.

## 2 Materials and methods

### 2.1 Materials

PLGA with composition 50 : 50, acid terminated with molecular weight of 7000–17 000 a.m.u., Rhodamine 6G (R6G), poly(vinyl alcohol) (PVA) with an average molecular weight of ∼31 000 a.m.u. (86.7–88.7 mol% hydrolysis), *O*,*O*′-bis(2-aminopropyl) polypropylene glycol-*block*-polyethylene glycol-*block*-polypropylene glycol (PEG) with an average molecular weight of 1900 a.m.u., *N*-(3-dimethylaminopropyl)-*N*′-ethylcarbodiimide (EDC), and *N*-hydroxysuccinimide (NHS) were purchased from Sigma Aldrich, Inc. (St. Louis, MO). Dichloromethane (CH_2_Cl_2_) was purchased from Fisher Scientific Inc. (Fair Lawn, NJ).

### 2.2 Preparation of PLGA nanoparticles

R6G was dispersed into PLGA nanoparticles (R6G-PNP) by following a single emulsion – solvent evaporation technique.^28,29^ Briefly, 50 mg of PLGA and 2.5 mg of R6G were dissolved in 5 mL of CH_2_Cl_2_. Next, 10 mL of 5% PVA aqueous solution was added to the organic phase. The mixture was emulsified in an ice bath with a XL2020 ultrasonicator (Misonix Inc., Farmingdale, NY, USA) operating at 55 W power output for one minute to obtain a single emulsion system. The emulsion was later broken by evaporation of the organic solvent using a water bath at 37 °C and 120 rpm during 6 h. Next, the solution was washed with three centrifugation cycles at 4900 rcf for 30 min using an IEC Centra-4B centrifuge (International Equipment, Inc., Nashville, TN, USA). During the first two centrifugation cycles, the supernatant was removed, and the precipitate was resuspended in 10 mL of distilled water. Finally, the resulting nanoparticles (R6G-PNP) were resuspended in 5 mL of 10 mM PBS buffer pH 7.4. All experiments were performed by triplicate.

### 2.3 Nanoparticle PEGylation

To conjugate PEG to the polymeric nanoparticles, the previously freshly prepared R6G-PNP solution was mixed with 0.0714 mmol of EDC and 0.0714 mmol of NHS. Then, 0.0714 mmol of PEG was added to the mixture. The reaction solution reacted for 2 hat room temperature to allow PEG coupling. Afterward, the prepared nanoparticle hybrids R6G-PNP-PEG were purified by two cycles of centrifugation at 4900 rcf for 30 min each one and then particles was freeze-dried for further use.

### 2.4 Nanoparticle characterization

The morphological characteristics of R6G-PNP-PEG were observed using a S-4800 field emission scanning electron microscope (Hitachi Corporation, Tokyo, Japan). Samples were prepared by their immobilization onto carbon-coated 400-mesh copper grids (Ted Pella Inc., Redding, CA, USA). Particle size and zeta potential were measured with a Zetasizer Nano ZEN3600 particle size analyzer (Malvern Instruments, Westborough, MA, USA). Refraction index used in the analysis was 1.33 and 10 mM sodium phosphate buffer pH 7.4 was used as the dispersant. The nanoparticle dispersions were diluted with the same buffer until getting a number of counts that allow a good signal to noise ratio, but enough to prevent multiple scattering from happening. Size analysis of each sample consisted of an average of 10 measurements. Zeta potential of each sample was measured by duplication with at least ten runs at constant temperature (25 °C) by laser Doppler electrophoresis. Z-averages and zeta potentials were obtained from three independent experiments. Drug loading and encapsulation efficiency were determined by first dissolving a known mass of R6G-PNP-PEG with 1 M sodium hydroxide, followed by the addition of hydrochloric acid to equilibrate the pH of the solution at 7.4. R6G content in the dissolved nanoparticle suspension was analyzed by absorbance measurements at 524 nm using a spectrophotometer UV-1800 (Shimadzu Co., Ltd., Japan). The concentration of R6G was determined by standard calibration curves in 10 mM PBS buffer pH 7.4. Drug loading (DL) was determined as the mass ratio of R6G entrapped in R6G-PNP-PEG to the mass of R6G-PNP-PEG recovered.^30^ Encapsulation efficiency (EE) was determined as the mass ratio of R6G entrapped in R6G-PNP-PEG to the theoretical maximum loading, a value considered when entire supplied R6G is encapsulated in nanoparticles.^31^

### 2.5 Experimental drug release

R6G release from R6G-PNP-PEG nanoparticle suspensions was determined by a dialysis membrane method under sink conditions.^28,32–34^ Release experiments were conducted at three different temperatures: 37 °C, 47 °C, and 57 °C. R6G-PNP-PEG samples dispersed in 3 mL of 10 mM sodium phosphate buffer pH 7.4 were placed into a dialysis membrane of cellulose with 12 000–14 000 molecular weight cut-off (Spectrum Laboratories, Inc., Rancho Dominguez, CA, USA), and incubated in 30 mL of 10 mM sodium phosphate buffer pH 7.4 at a controlled temperature. At time intervals ranging from 0 to 27 days, 1 mL samples were withdrawn from the incubation medium and analyzed for content of R6G by spectrophotometry by measuring their absorbance at 524 nm and correlated to the corresponding calibration curve. Each volume withdrawn was replenished with 1 mL of 10 mM sodium phosphate buffer pH 7.4. The mass of R6G from the withdrawn volumes was considered in the release profiles with a mass balance. Solutions of free R6G (not encapsulated in nanoparticles) were also analyzed by this dialysis membrane method as controls at the same conditions described above. This analysis was performed to determinate is the dialysis membrane mass transfer resistance was significant in the experimental design.

### 2.6 Drug release analysis

Drug release analysis involves at least one mechanism of release. This mechanism might consist of two possible scenarios, one that considers transport of the drug from the matrix to the media and another one that involves the dissolution of the matrix as well. To contemplate the dissolution of the matrix and the transport of the drug in the drug release analysis from the biodegradable matrix, like PLGA nanoparticles, more than one mechanism of release should be considered. A quite comprehensive review for the release of drugs from nanoparticles that considers several physical factors that affect the different mechanism stages of drug release in PLGA nanoparticle delivery systems can be found in the literature.^5^ One of the most important issues should be the temperature of the drug release system. It is well known that temperature plays an active role in the solubility of chemical moieties and the processes of dissolution and hydrolysis of biodegradable compounds, like PLGA. Dunne *et al.*^7^ reported that the rate of PLGA degradation increases proportionally with increasing incubation temperature. In the present work, the release of drugs from biodegradable nanoparticles was analyzed using two different coupled models of release that considers the simultaneous contribution of two or three mechanism stages of release. The mechanisms considered are initial burst, nanoparticle degradation–relaxation, and diffusion of the drug. The initial burst was analyzed by considering the release of drugs as an interfacial diffusion process between the spherical nanoparticle and liquid media, as described in the literature.^27^ This phenomenon considers that there is no drug dissolved at an initial time, thus resulting in the following first-order equation:1
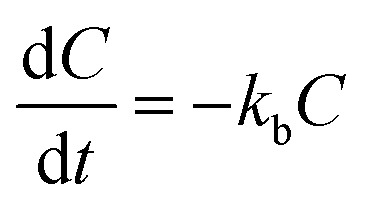
where *k*_b_ is the initial burst constant, and *C* is the concentration of the drug in the release environment at time *t*. A solution of eqn (1) is obtained by considering that at the initial time the concentration of drug in the sphere is equal to the initial mass of solute per volume of sphere. Also, the mass released at time *t* is obtained from the difference between the initial mass in the sphere and the remaining mass in the sphere at that time *t*. The solution of eqn (1) results in the equation:2
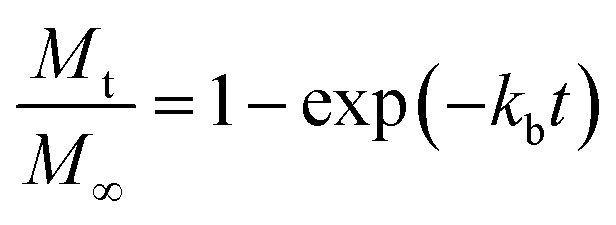
where *M*_t_ is the cumulative amount of drug released at time *t*, and *M*_∞_ is the cumulative amount of drug released at the infinite time. In the proposed model, in this initial burst stage, the temperature dependency was incorporated in the initial burst constant *k*_b_ and described by an equation of the Arrhenius form:3
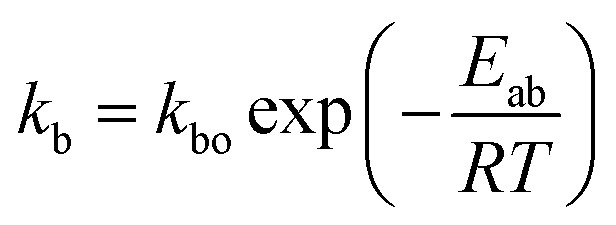
where *k*_bo_ is a constant, *E*_ab_ is the energy of activation for the burst phase, *R* is the gas constant (cal deg^−1^ mol^−1^), and *T* is the absolute temperature.

The second mechanism of release considered in the analysis is the nanoparticle degradation–relaxation. This phenomenon is related to polymer degradation using the hydrolysis of the PLGA in the liquid media. The nanoparticle interphase with liquid is the first portion of the nanoparticle exposed to hydrolysis, and thus influenced by surface area, composition and molecular weight of the polymer, as reported in the literature.^27^ This mechanism of release has been described with an analogous form of the equation of Prout–Tompkins:^20,21^4
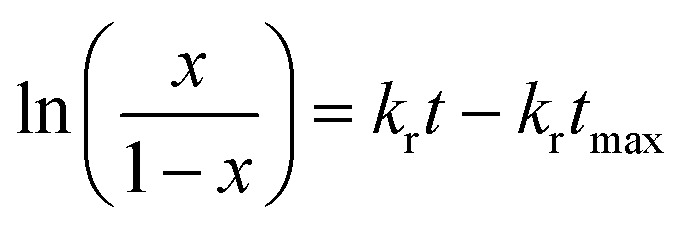
where *x* is the fractional mass released at time *t*; and equivalent to fractional release *M*_t_/*M*_∞_, *k*_r_ is the rate of degradation–relaxation constant, and *t*_max_ is the time to maximum rate of drug release or time to achieve 50% of release.^35^ Rearranging eqn (4) results in the equation,5
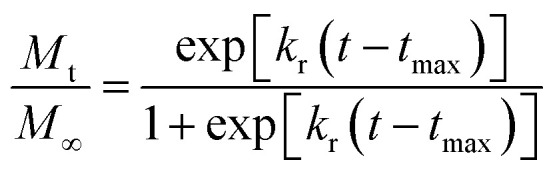


In this work, the degradation–relaxation constant, *k*_r_, as a function of temperature was also evaluated using the Arrhenius equation form:^7^6
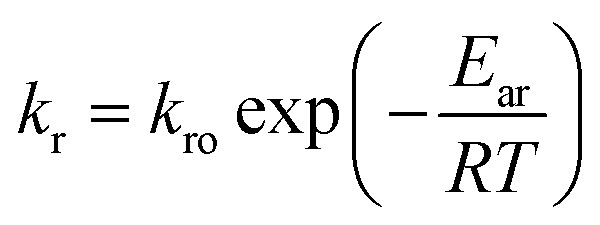
where *k*_ro_ is a constant, *E*_ar_ is the energy of activation, *R* is the gas constant (cal deg^−1^ mol^−1^), and *T* is the absolute temperature. Similarly, *t*_max_ was incorporated as a temperature dependent parameter using an Arrhenius equation expression:7
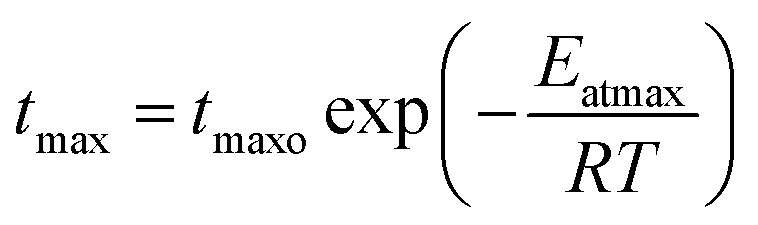
where *t*_maxo_ is a constant, *E*_atmax_ is the energy of activation, *R* is the gas constant (cal deg^−1^ mol^−1^), and *T* is the absolute temperature.

The last mechanism of release proposed in this work is a drug release step carried out by fickian diffusion. The diffusion stage was evaluated with a general mass balance that considers the drug release, because of symmetry, only in radial coordinates. Thus, in the model, the concentration of the drug is uniform at a fixed radius, the effective diffusion coefficient (*D*_e_) is considered independent of time and position, and no chemical reaction or decomposition of the drug occurs in the system.^27^ The governing equation that results from these features is given by the transient concentration profile of the drug in spherical coordinates with the only contribution of diffusion in the radial direction:8
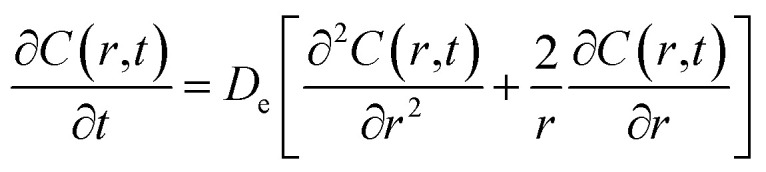
where concentration (*C*) is a function of time and radial position in the nanoparticle. The boundary conditions considered in the model are symmetry at the center of the sphere (eqn (9)), and that the concentration of drug on or close to the surface of the sphere is negligible for times more significant than zero, since concentration is lower than the solubility limit of the drug, *r*_1_ is the nanoparticle radius (eqn (10)). The initial condition considered in the model is that at time equal zero, all the encapsulated drug is homogeneously distributed over the entire volume of the sphere (*v*_s_) (eqn (11)).9
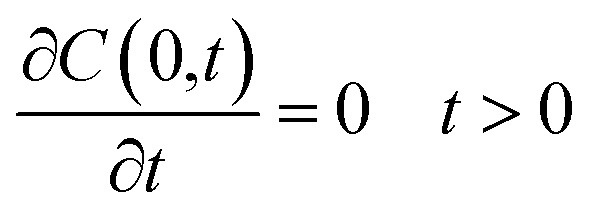

10 *C*(*r*_1_,*t*) = 0 *t* > 0

11
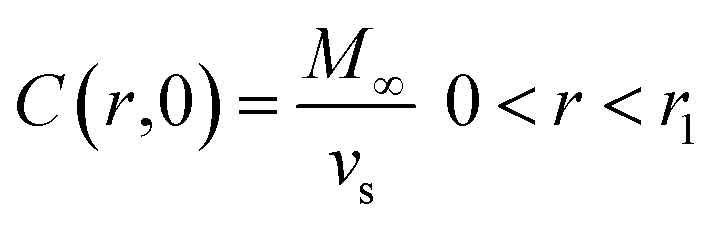


An analytical solution for eqn (8) by separation of variables with these initial and boundary conditions is given by eqn (12).12
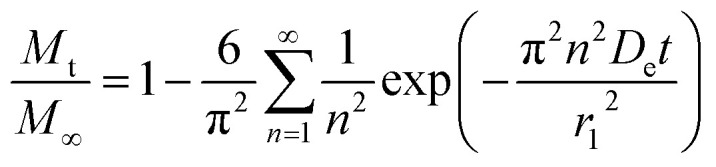


For the particular model presented here, the temperature dependence in this diffusion stage was also incorporated in the diffusion coefficient constant using similarly the Arrhenius equation expression. The resulting equation is thus given by:13
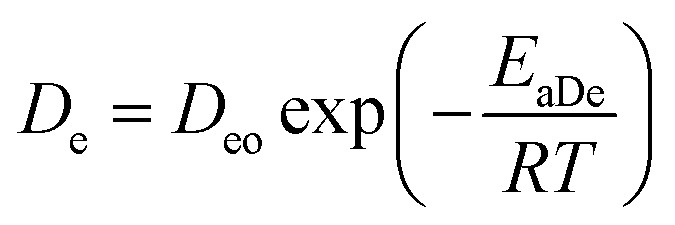
where *D*_eo_ is a constant, *E*_aDe_ is the energy of activation, *R* is the gas constant (cal deg^−1^ mol^−1^), and *T* is the absolute temperature. An explanation of *E*_aDe_ could be in terms of the energy needed for the diffusion through water-filled pores, diffusion through the polymer matrix, to overcome the polymer–drug interactions, drug–drug interactions, among others. Thus, at this point, the three mechanisms of release involved in this analysis contain temperature dependent parameters and could be examined independently in different release systems. Drug release from biodegradable nanoparticles usually follows a biphasic or a triphasic release profile behavior. Experimental drug release data were analyzed first by a biphasic model that combines initial burst, eqn (2) and particle degradation, eqn (5) with temperature dependency in the corresponding parameters, the Model BR. This model of release represents contributions over the total drug release profile with a first order initial burst and the nanoparticle degradation–relaxation. The combined equation can be expressed as:14
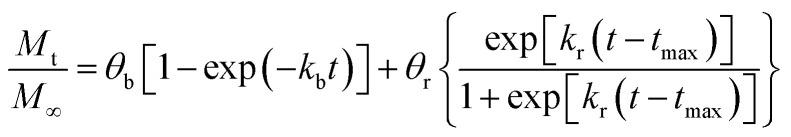
where *θ*_b_ is the contribution of initial burst release over total mass drug release. Model BR was adjusted to experimental data of release at the three temperatures by a nonlinear least-squares algorithm in software MATLAB® (MathWorks, USA) to obtain the parameters: *θ*_b_, *k*_b_, *k*_r_, and *t*_max_. The parameters derived from the fitting at each temperature of release were used to get the values of the initial burst constant (*k*_b_) in eqn (3), the rate of degradation–relaxation constant (*k*_r_) in eqn (6), and the time to maximum rate of drug release or the time to achieve 50% of release (*t*_max_) in eqn (7) respectively.

The second model or Model BRD was obtained by adding fickian diffusion contribution to Model BR, in other words, it represents the linear combination of eqn (2), eqn (5), and eqn (12). The equation that results from the coupling of these three mechanisms of release is:15
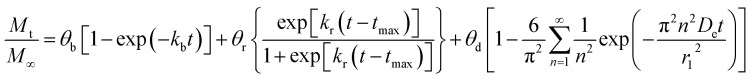
where *θ*_b_, *θ*_r_, and *θ*_d_ are the contribution fractions of initial burst, nanoparticle degradation–relaxation, and diffusion mechanisms, respectively, over the total mass drug release. The relation *θ*_b_ + *θ*_r_ + *θ*_d_ = 1 was introduced to the analysis to introduce mathematical consistency. The Model BRD composed by the linear system showed in eqn (15) contains six unknown parameters (*θ*_b_, *θ*_r_, *k*_b_, *k*_r_, *t*_max_, and *D*_e_), since *θ*_d_ = 1 − *θ*_b_ − *θ*_r_. Unknown parameters were determined by adjusting the experimental data by a nonlinear least-squares algorithm in software MATLAB® (MathWorks, USA), similarly to the analysis for model Model BR. Effects of temperature over initial burst constant (*k*_b_), the rate of degradation–relaxation constant (*k*_r_), time to achieve 50% of release (*t*_max_), and effective diffusion (*D*_e_) were evaluated using eqn (3), eqn (6), eqn (7), and eqn (13), respectively.

To compare more effectively both release models (Model BR and Model BRD) and since they contain a different number of parameters, in addition to using the coefficient of determination (*R*^2^), an adjusted coefficient of determination (*R*_adjusted_^2^) was incorporated in the analysis. This coefficient is given by the equation,16
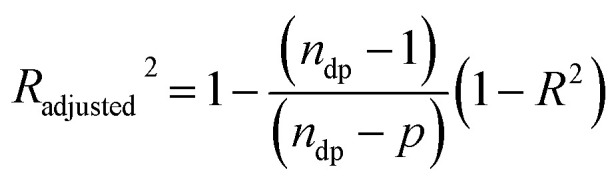
where *n*_dp_ is the number of data points (*M*_t_/*M*_∞_), and *p* is the number of parameters in the model. Whereas the *R*^2^ always increases or stays constant when adding new model parameters, *R*_adjusted_^2^ can actually decrease, thus indicating if the new parameter really improves the model or it might lead to overfitting.^36^

## 3 Results and discussion

### 3.1 PLGA nanoparticles preparation and PEGylation

**Fig. 1:**
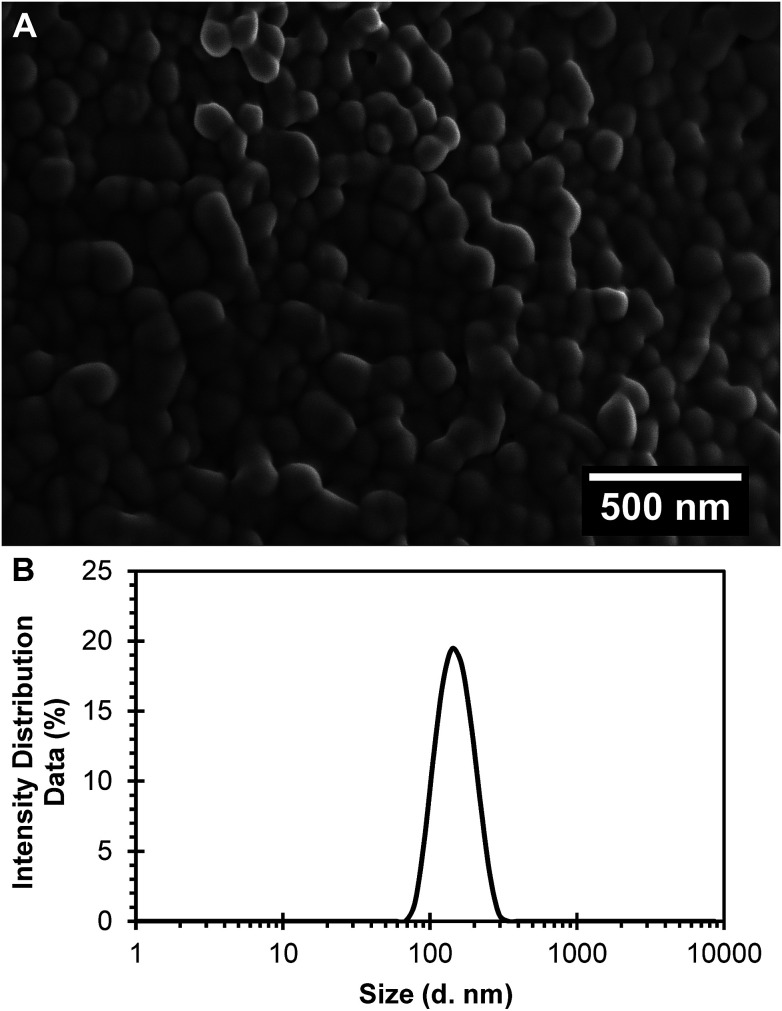
Characterization of the R6G loaded PEGylated nanoparticles (R6G-PNP-PEG). (A) Scanning electron microscopy images show a smooth surface for nanoparticles. (B) Dynamic light scattering spectra R6G-PNP-PEG were used to determine the average diameter and polydispersity index of nanoparticles.

R6G was characterized by spectrophotometry at 524 nm by running a standard curve in PBS buffer pH 7.4 (molar extinction coefficient, *ε* = 51 614 M^−1^ cm^−1^). Polymeric nanoparticles prepared with 5% of theoretical drug loading of R6G following a single emulsion-solvent evaporation technique, and after PEGylation to obtain PEGylated (R6G-PNP-PEG) resulted in a 0.59% drug loading and 11.71% encapsulation efficiency. Nanoparticles had smooth surfaces as depicted by scanning electron microscopy (Fig. 1A). Dynamic light scattering spectra for R6G-PNP-PEG gave an average diameter size of 142 ± 0.14 nm, a polydispersity index of 0.062 ± 0.016 (Fig. 1B), and a zeta potential of −22.7 ± 1.3 mV. These values indicate that particles are monodisperse and with good stability in suspension in PBS buffer.

### 3.2 Drug release studies

**Fig. 2:**
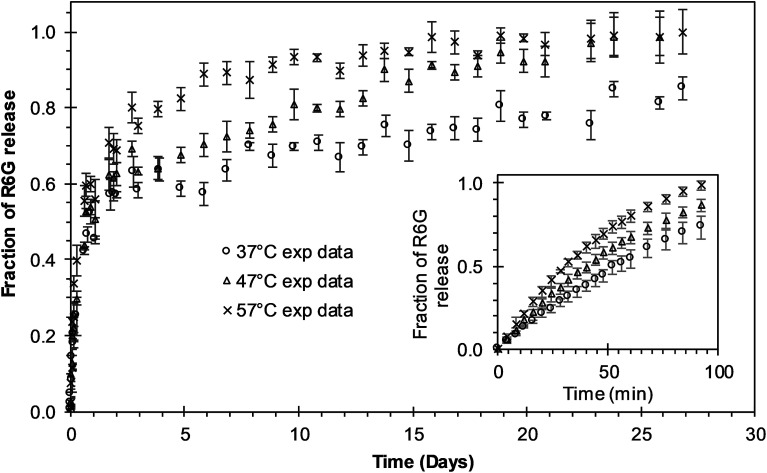
Experimental release of R6G from PEGylated nanoparticles at different temperatures. The insert shows the free R6G release from the dialysis membrane. Circles represent the release at 37 °C; triangles represent the release at 47 °C; “*x*” points represent the release at 57 °C.

Experimental release of R6G from PEGylated nanoparticles was evaluated at three different temperatures during 27 days to analyze the different phases of release. During the first six days of release at 37 °C, the curve presents a release phase characteristic of an initial burst. After that initial phase, nanoparticle degradation–relaxation takes place together with a diffusion stage to describe the complete release profile (Fig. 2). Release at 47 °C shows as well, a multiphasic behavior that reflects initially to the first burst effect, followed by the second stage of particle relaxation or polymer degradation and finally a phase due to diffusion (Fig. 2). At this temperature, however, the duration of the initial burst was around four days. In the experimental data at 57 °C (Fig. 2) the duration of the initial burst stage is smaller compared with the initial burst observed at 37 °C and 47 °C, indicating a clear effect of temperature over drug release rate. From these results, we infer that the overall rate of release is directly proportional to temperature. These results indicate that in general temperature has significant effects over parameters of release. Increments of temperature could lead to increments in the solubility of the drug and the rate of degradation of PLGA and therefore in the overall drug release rates. These results corroborate previous studies where the rate of polymer degradation increases by increasing incubation temperature.^7^ Free R6G was used to analyze the possible effects of the dialysis membrane transport resistance. R6G transferred through the dialysis membrane shows that membrane transport resistance is practically negligible; for the three different incubation temperatures analyzed, almost all the R6G placed inside the membrane was released to the media within 90 minutes, as could be depicted in the insert of Fig. 2. This result corroborates that experimental R6G release profiles presented in Fig. 2 correspond to the drug released from the nanoparticles, neglecting the mass transfer effects from the dialysis membrane.

### 3.3 Drug release analysis

**Fig. 3:**
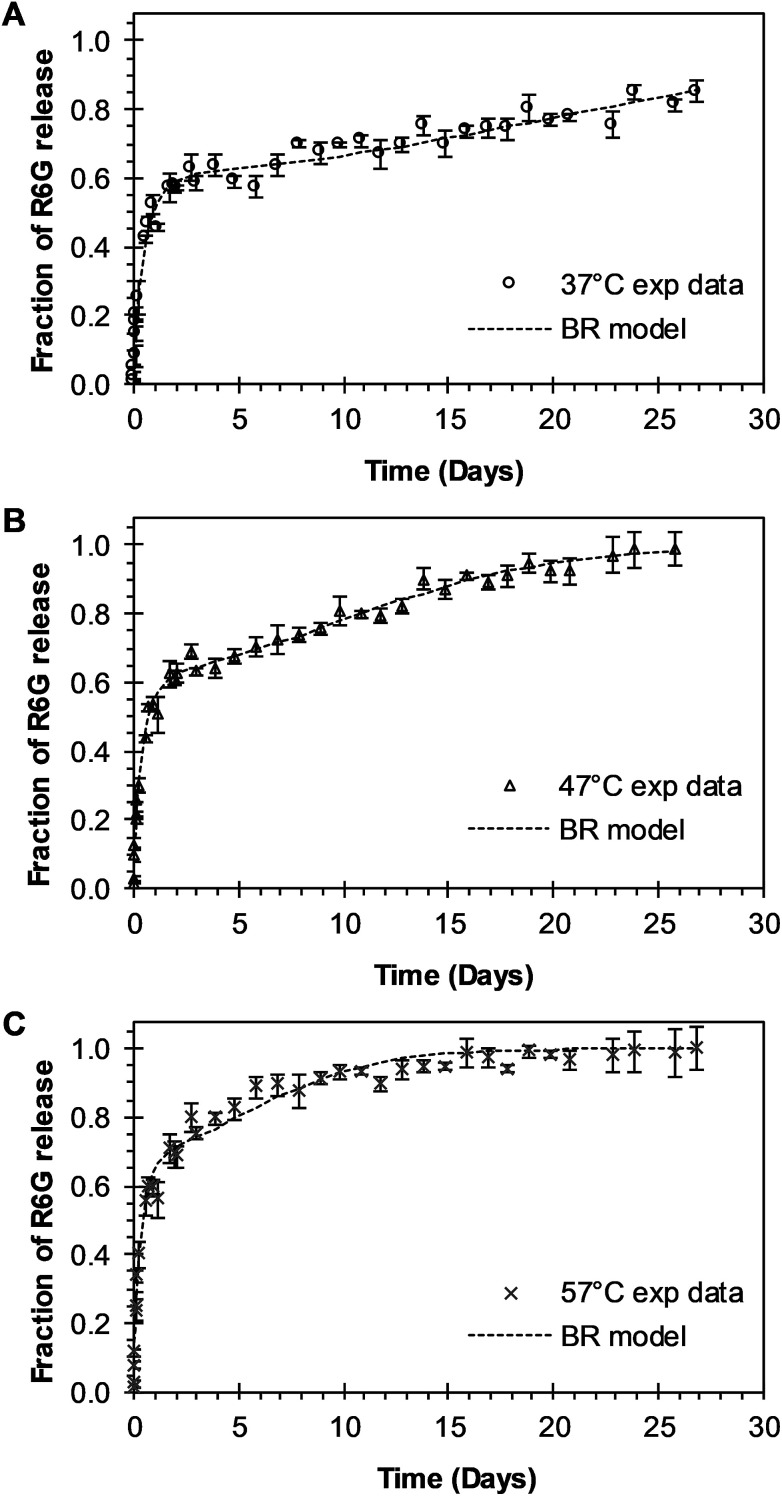
Experimental and theoretical R6G release profile from PEGylated nanoparticles at different temperatures. (A) Circles represent experimental data at 37 °C, and the square dot line represents fitting to the experimental data when BR model of eqn (14) was applied (*θ*_b_ = 0.5567). (B) Triangles represent experimental data at 47 °C, and the square dot line represents eqn (14) fitting to experimental data when *θ*_b_ = 0.5453. (C) “*x*” points represent experimental data at 57 °C, and square dot line represents eqn (14) fitting to experimental data when *θ*_b_ = 0.6139.

**Fig. 4:**
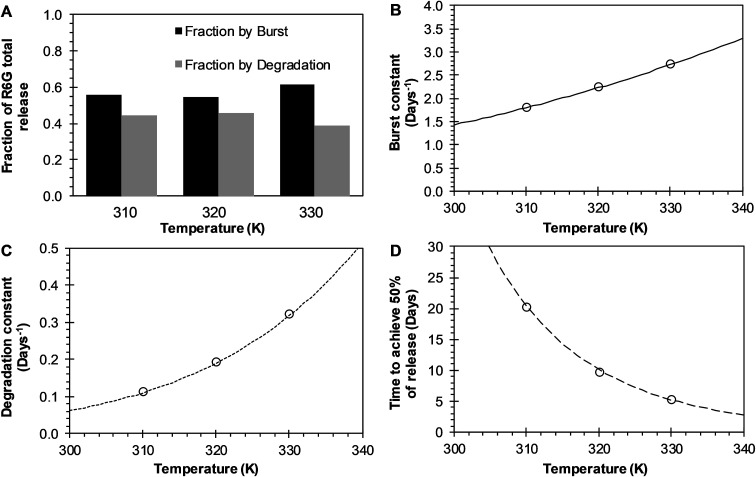
Effects of temperature over release parameters of R6G release from PEGylated nanoparticles when BR model is used. (A) The contribution of initial burst (black bar) and nanoparticle degradation–relaxation (dark gray) overall drug release phenomena. (B) Initial burst constant (circles), and the continuous line represents the fitting of Arrhenius equation when *k*_bo_ = 1703.0412 days^−1^ and *E*_ab_ = 4.2211 kcal mol^−1^. (C) Relaxation constant (circles), and square dot line represents the fitting of Arrhenius equation when *k*_ro_ = 4 921 997.7158 days^−1^ and *E*_ar_ = 10.7711 kcal mol^−1^. (D) Time to achieve the 50% of release (circles), and long dash line represents the fitting of the Arrhenius equation when *t*_maxo_ = 3.3883 × 10^−9^ days, and *E*_atmax_ = −13.8709 kcal mol^−1^.

BR Model Analysis. The BR model given by eqn (14) was fitted to the R6G release data from the PEGylated nanoparticles, and the resulting parameters are presented in Table 1. The Model BR considers the simultaneous effect of initial burst and nanoparticle degradation–relaxation, providing adequate fit with all the drug release experimental data at temperatures of 37 °C, 47 °C, and 57 °C as seen in Fig. 3A–C. By analyzing the contributions of each mechanism of release (Fig. 4A), it can be observed that the initial burst and nanoparticle degradation relaxation contributions over the total drug release are almost constant for a temperature increase from 37 °C to 47 °C. When the temperature increases from 47 °C to 57 °C, the initial burst contribution increases from 0.54–0.61, and as a consequence the nanoparticle degradation–relaxation decreases proportionally, as is shown in Fig. 4A. The release analysis with the Model BR in the range of temperature considered shows that factors affecting the transport of the drug to the liquid media, such as drug solubility are more sensitive to the increments of temperature than the degradation of the nanoparticles. Thus, with a temperature increase, the solubility of the drug increases providing a larger effect on the initial burst release. This increase will be reflected on the value of its respective parameter and becomes larger than the nanoparticle's degradation parameter, as shown in Fig. 4B. Also, previous works suggest that the amount of drug released during burst is mostly influenced by the formulation characteristics and the synthesis parameters, whereas the drug release kinetics is also influenced by the molecular properties of the drug.^37^ The linearization of eqn (3), by plotting ln *k*_b_*versus* the values of the reciprocal temperature, was used to obtain the constant values of *k*_bo_ and *E*_ab_. The slope of the curve was taken as *E*_ab_/*R* and the exponential of the intersection as *k*_bo_, and the results given in Table 1. The dependency of the burst constant shown in Fig. 4B obtained with eqn (14) against experimental data are represented by circles and the solid line denotes the prediction of the burst constant using eqn (3) in the given temperatures range. In figure Fig. 4A it is also shown the corresponding effect on the degradation phase fraction when changes in burst fraction occur at different temperatures. It is known that the degradation of PLGA nanoparticles degradation takes place by systematic degradation of monomers first on the nanoparticle surface due to water uptake that subsequently promotes further polymer degradation.

**Table 1:** Parameters of R6G release from PEGylated nanoparticles at different temperatures. Parameters were determined and used in the mathematical development of BR model

Parameters	Description	Unit	Release temperature (°C)		
			37	47	57
*k*_b_	Burst constant	days^−1^	1.8053	2.2306	2.7337
*k*_bo_	Arrhenius constant (burst)	days^−1^	1703.0412		
*E*_ab_	Energy of activation (burst)	kcal mol^−1^	4.2211		
*θ*_b_	Fraction of burst release	—	0.5567	0.5453	0.6139
*k*_r_	Degradation–relaxation constant	days^−1^	0.1109	0.1924	0.3198
*k*_ro_	Arrhenius constant (degradation)	days^−1^	4 321 997.7158		
*E*_ar_	Energy of activation (degradation)	kcal mol^−1^	10.7711		
*t*_max_	Time to achieve 50% of release	days	20.1849	9.5761	5.1626
*t*_maxo_	Arrhenius constant (*t*_max_)	days	3.3883 × 10^−9^		
*E*_atmax_	Energy of activation (*t*_max_)	kcal mol^−1^	−13.8709		
*θ*_r_	Fraction of NP relaxation release	—	0.4433	0.4547	0.3861
*R* ^2^	Coefficient of determination	—	0.9929	0.9963	0.9931
*R*_adjusted_^2^	Adjusted coefficient of determination	—	0.9923	0.9960	0.9925

Temperature has been used to enhance such degradation, and proportionally it has been observed that an increase in temperature increases in general, the release rate of drugs. The degradation–relaxation constant dependence with temperature was analyzed with the Arrhenius correlation with eqn (6). Similarly to the analysis of the burst constant values, the parameters of the equation *k*_ro_ and *E*_ar_ were determined by plotting the rates of relaxation in a logarithm form as (ln *k*_r_) *versus* the reciprocal temperature. Similar effects of temperature are observed in this case with the degradation constant values concerning the behavior of the initial burst constant at the experimental values of 37 °C, 47 °C and 57 °C, as shown in Fig. 4C. The circles and dot line in Fig. 4C represents the fitted degradation constant given by eqn (14), and the predicted values of the degradation constant given by eqn (6), respectively. Fig. 4D shows how significant the effects of temperature could be over this constant when determining the time to achieve the 50% of drug release. This parameter decreases significantly and inversely proportional with temperature as shown in Fig. 4D. In the absence of this correlation, it would be quite difficult to assess such temperature dependency. Here, the temperature dependence was analyzed with equation eqn (7) where once more the parameters *t*_maxo_ and *E*_atmax_ were determined by plotting the natural logarithm values of rates of the time to achieve the 50% of release (ln *t*_max_) *versus* reciprocal temperature. The circles in Fig. 4D, represent the fitted time to achieve 50% of release using eqn (14), and the long dash line represents a prediction of the time to achieve 50% of release using eqn (7). This result could be explained coupling the effects of temperature over the degradation of the nanoparticles such that an increase in temperature, the nanoparticles degrade proportionally to that increase, and such degradation allows for a proportional faster drug release.

#### 3.3.1 BRD model analysis

**Fig. 5:**
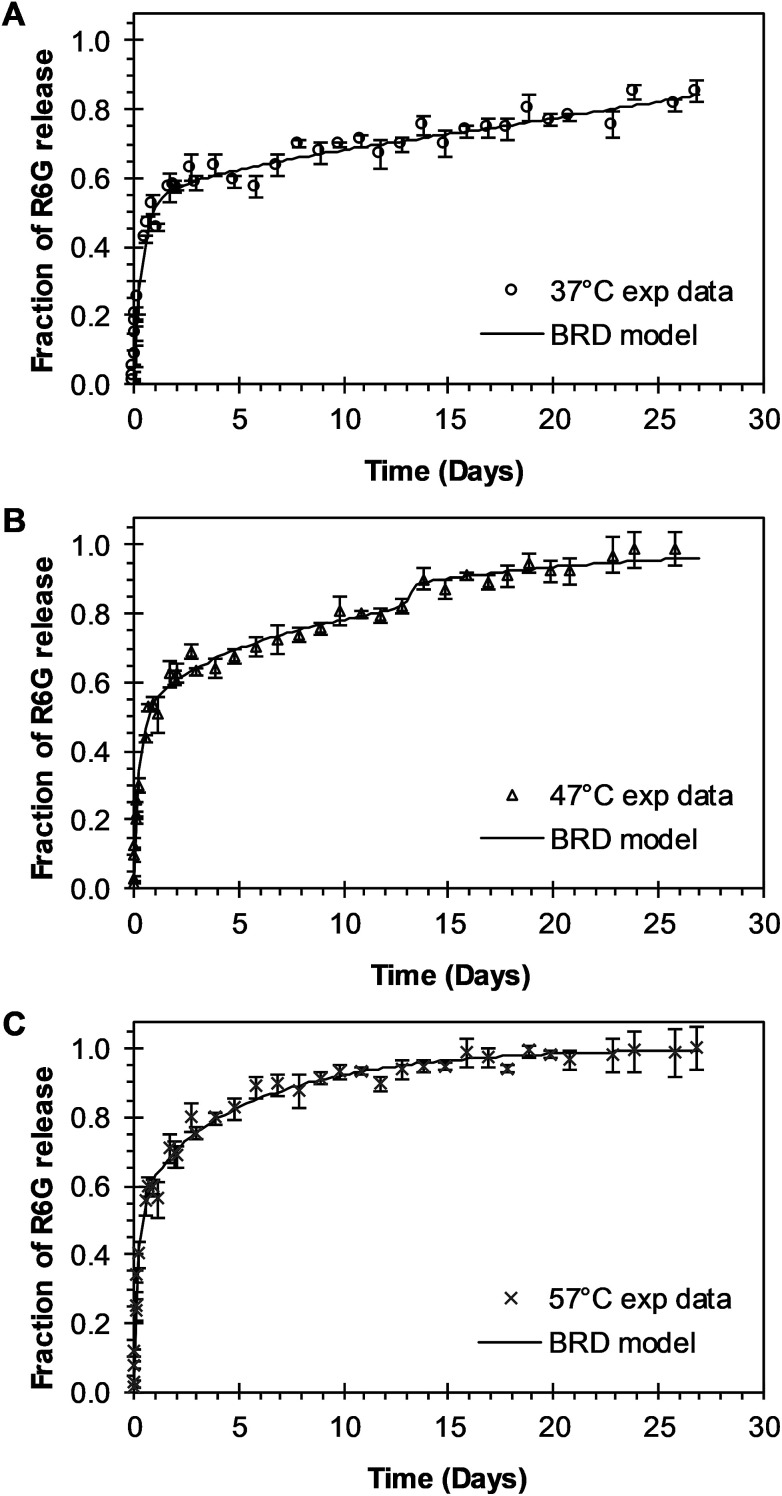
Experimental and theoretical R6G release profile from PEGylated nanoparticles at different temperatures. (A) Circles represent experimental data at 37 °C, and the continuous line represents fitting to experimental data when BRD model of eqn (15) was applied (*θ*_b_ = 0.4636; *θ*_r_ = 2372). (B) Triangles represent experimental data at 47 °C, and the continuous line represents eqn (15) fitting to experimental data when *θ*_b_ = 0.3988 and *θ*_r_ = 0.0688. (C) “*x*” points represent experimental data at 57 °C, and continuous line represents eqn (15) fitting to experimental data when *θ*_b_ = 0.3945 and *θ*_r_ = 2.6144 × 10^−9^.

**Fig. 6:**
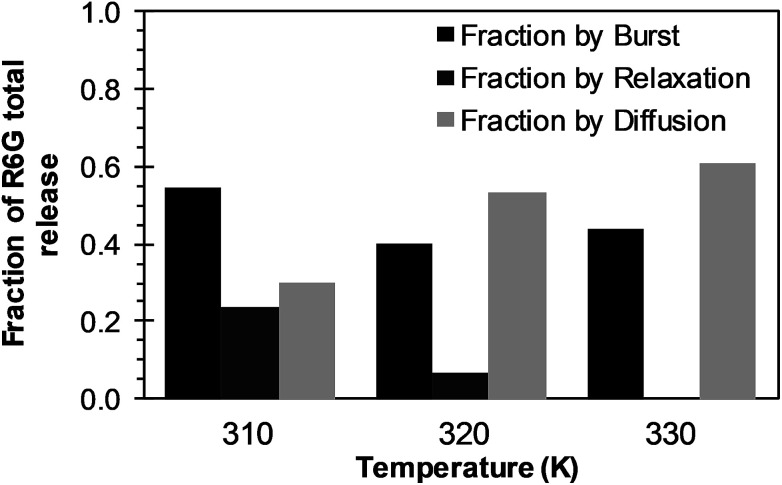
Effects of temperature over R6G release contributions of initial burst (black bars), nanoparticle degradation–relaxation (dark gray bars), and diffusion (light bars), when BRD model is applied.

**Fig. 7:**
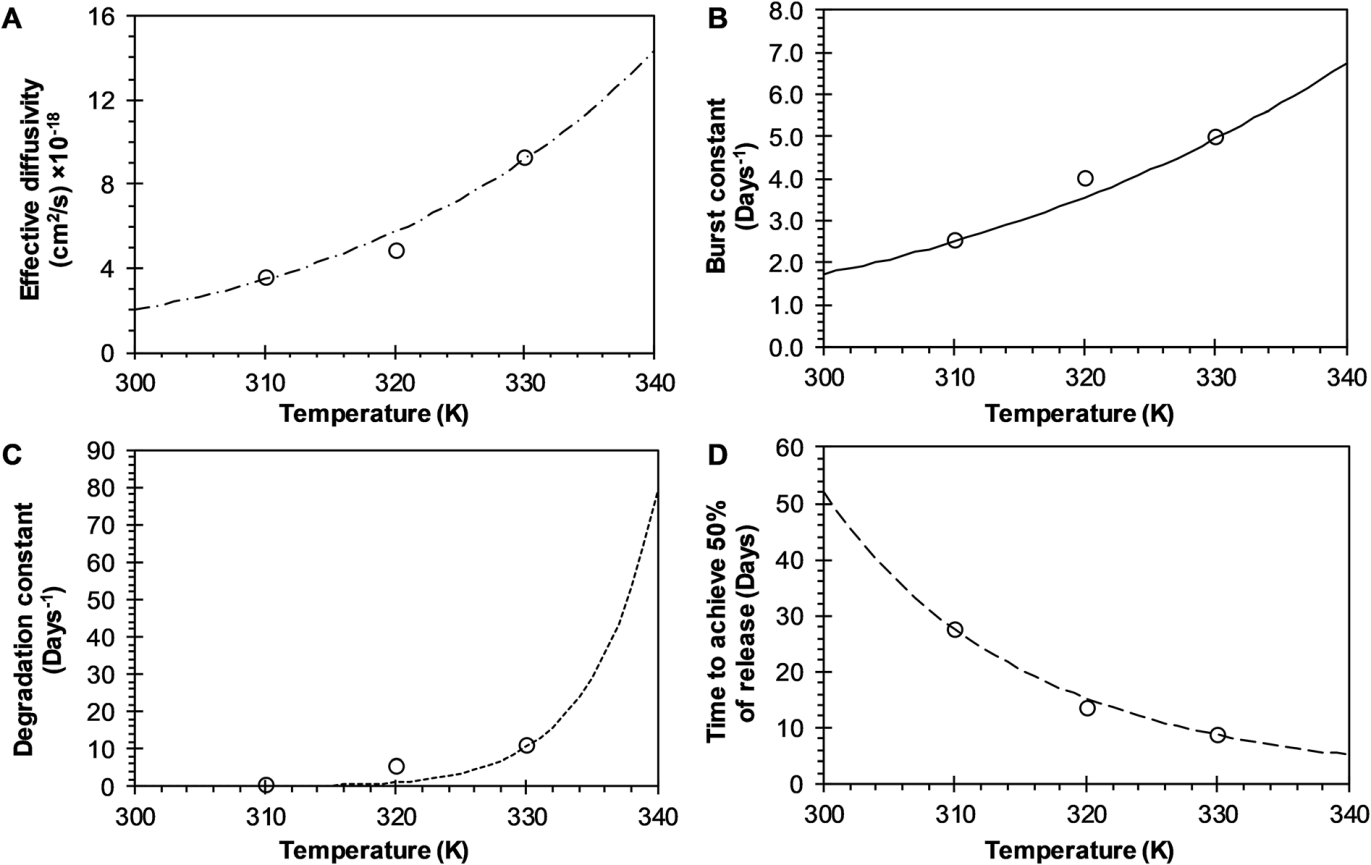
Effects of temperature over release parameters of R6G release from PEGylated nanoparticles when BRD model is used. (A) Effective diffusion coefficient (circles), and slash-dot line represents fitting of Arrhenius equation when *D*_eo_ = 3.3345 × 10^−11^ cm^2^ s^−1^ and *E*_aDe_ = 9.9030 kcal mol^−1^. (B) Initial burst constant (circles), and the continuous line represents fitting to Arrhenius equation when *k*_bo_ = 182 668.5606 days^−1^ and *E*_ab_ = 6.8957 kcal mol^−1^. (C) Relaxation constant (circles), and square dot line represent fitting of Arrhenius equation to these points when *k*_ro_ = 7.2268 × 10^30^ days^−1^ and *E*_ar_ = 45.0589 kcal mol^−1^. (D) Time to achieve the 50% of release (circles) and long dash line represent fitting of Arrhenius equation to these points when *t*_maxo_ = 1.3220 × 10^−7^ days, and *E*_atmax_ = −11.7986 kcal mol^−1^.

The analysis of the mechanisms of release that considers the initial burst, the nanoparticle degradation and drug diffusion phenomena was considered in the BRD model described in eqn (15). This model was fitted to the experimental data of R6G release from R6G-PLGA-PEG nanoparticles. The resulting parameters from the fitting are summarized in Table 2. From Fig. 5A–C it can be observed that the BRD model [eqn (15)] fits quite well the experimental data during the entire drug release process for the three incubation temperatures analyzed. The contribution to the release process of each independent mechanism regarding the fraction of release is presented in Fig. 6. Here it can be observed that the temperature dependence analysis provides a much better idea of the intricate interdependence of stages involved in the processes of drug release phenomena. The release contribution from particle degradation decreases considerably with temperature increments while the contribution from the initial burst is maintained in the same order and the contribution to the drug release by diffusion increments proportionally with the temperature increase. The differences in these parameters values, when compared with results obtained with the BR model, indicate that mechanism of diffusion plays a significant role in the overall release behavior. Also, the step observed in the fitting of Fig. 5B could be explained in terms of the increase of the diffusion contribution with the temperature. Then, the transport of R6G through the nanoparticle increase with the temperature, and consequently, at higher temperatures more R6G is released by the diffusion mechanism. The temperature incorporation in mathematical models involving several stages of release should play an important role when analyzing experimental data. The substantial increments in the diffusion contribution as the temperature increase could be due to several factors. For example, an increase in the solubility of drugs increases the diffusivity of the drug from hydrophobic polymer matrices by decreasing the drug–polymer attraction. Effective diffusion coefficient shown in Fig. 7A (circles) was analyzed with equation eqn (13). The parameters of the equation *D*_eo_ and *E*_aDe_ were determined by plotting the natural logarithm of the effective diffusion rates (ln *D*_e_) against reciprocal temperature, and presented in Table 2. The slash-dot line in Fig. 7A represents the prediction of the effective diffusion using eqn (13). The values for effective diffusivity presented in Table 2 are in agreement to other reports for drug release of polymeric nanoparticles, and have been associated to drug–polymer interactions.^38^ Also, the values for effective diffusivity (Fig. 7A), the initial burst constant (Fig. 7B) and the degradation constant (Fig. 7C) increased with temperature. In the later case, it can be observed large value increments from the degradation constant concerning temperature increments. This significant tendency corroborates the effect of temperature over the degradation of nanoparticle discussed in Fig. 6. Also, the relaxation of the nanoparticle could be explained regarding the changes in the glass transition temperature of PLGA which is below 47 °C. Beugeling *et al.* found that in PLGA compacts the pulsatile release was greatly influenced by the *T*_g_ of the polymer.^39^ In Fig. 7B, the burst constant obtained from the fit of eqn (15) to experimental data is represented by circles and the solid line represents a prediction of the burst constant given by eqn (3) with the parameters *k*_bo_ and *E*_ab_ gave in Table 2. In both, the BR and the BRD models, an increase in values of initial burst constants are observed as the temperature increases. Similarly, the effect of temperature in the constant of degradation was also described with a relation of the Arrhenius form with equation eqn (6) and the parameters *k*_ro_ and *E*_ar_ were determined as described above. The circles and square dot line in Fig. 7C represents the fitted degradation constant from eqn (15) with the prediction values of this constant from eqn (6), respectively.

**Table 2:** Parameters of R6G release from PEGylated nanoparticles at different temperatures. Parameters were determined and used in the mathematical development of BRD model

Parameters	Description	Unit	Release temperature (°C)		
			37	47	57
*k*_b_	Burst constant	days^−1^	2.5244	3.9805	4.9721
*k*_bo_	Arrhenius constant (burst)	days^−1^	182 668.5606		
*E*_ab_	Energy of activation (burst)	kcal mol^−1^	6.8957		
*θ*_b_	Fraction of burst release	—	0.4636	0.3988	0.3945
*k*_r_	Degradation–relaxation constant	days^−1^	0.1274	5.2914	10.6848
*k*_ro_	Arrhenius constant (degradation)	days^−1^	7.22680605824739 × 10^30^		
*E*_ar_	Energy of activation (degradation)	kcal mol^−1^	45.0589		
*t*_max_	Time to achieve 50% of release	days	27.2835	13.1061	8.5548
*t*_maxo_	Arrhenius constant (*t*_max_)	days	1.3220 × 10^−7^		
*E*_atmax_	Energy of activation (*t*_max_)	kcal mol^−1^	−11.7986		
*θ*_r_	Fraction of NP relaxation release	—	0.2372	0.0688	2.6144 × 10^−9^
*D*_e_	Effective diffusion coefficient	cm^2^ s^−1^	3.4908 × 10^−18^	4.7670 × 10^−18^	9.2404 × 10^−18^
*D*_eo_	Arrhenius constant (diffusion)	cm^2^ s^−1^	3.3345 × 10^−11^		
*E*_aDe_	Energy of activation (diffusion)	kcal mol^−1^	9.9030		
*θ*_d_	Fraction of diffusion release	—	0.2992	0.5324	0.6055
*R* ^2^	Coefficient of determination	—	0.9889	0.9931	0.9944
*R*_adjusted_^2^	Adjusted coefficient of determination	—	0.9872	0.9920	0.9936

**Fig. 8:**
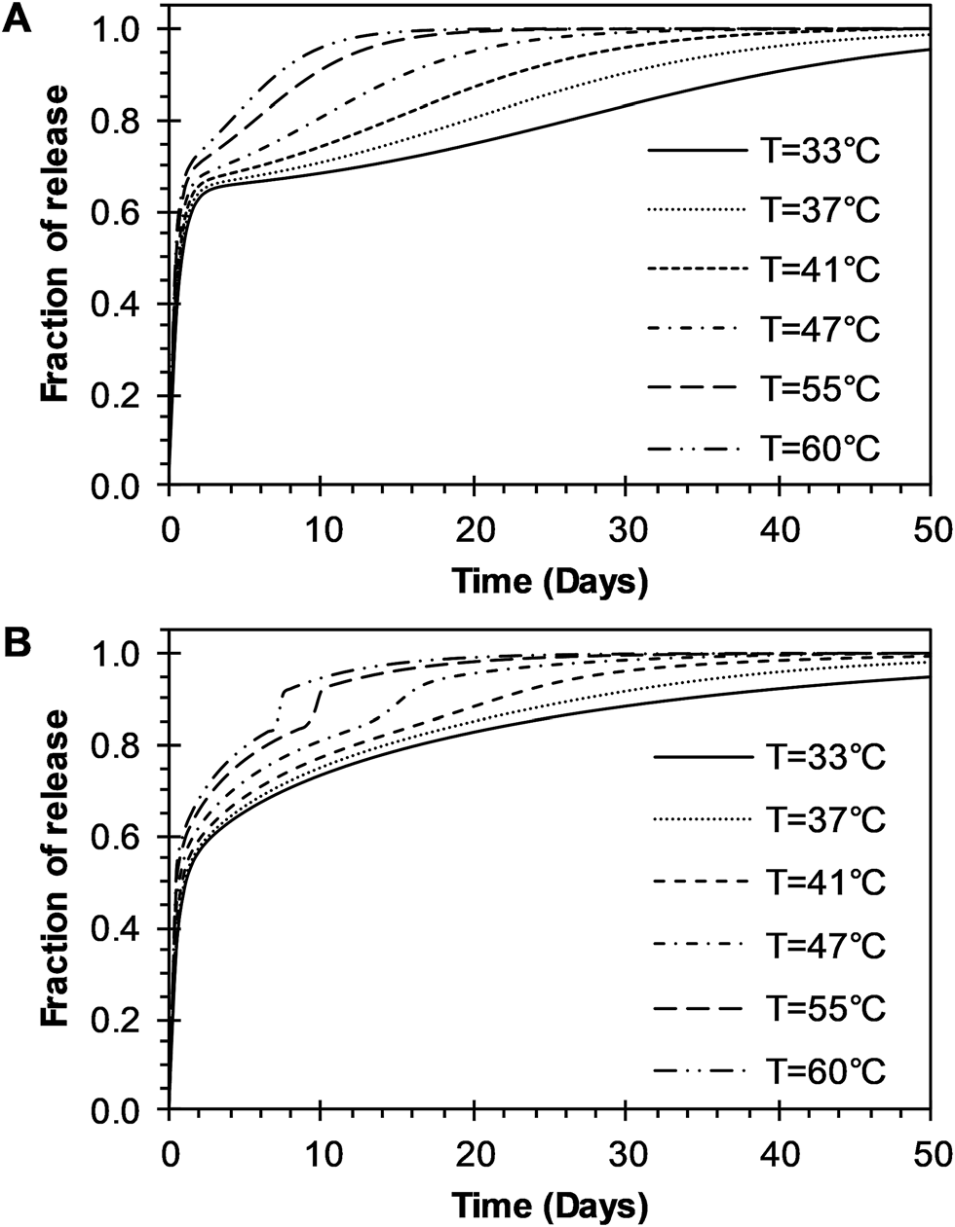
Parametric analysis of the effects of temperature over the R6G release from PEGylated nanoparticles using the two models developed in this work. (A) BR model presented in eqn (14). (B) BRD model given in eqn (15).

The time constant to achieve 50% of release was also evaluated with the BRD model. The resulting constant values were also obtained from the fitting of eqn (15) to experimental data and given in Table 2. The constant *t*_max_ decreases inversely proportional to temperature, as shown in Fig. 7D (circles). The dependence of *t*_max_ with temperature was analyzed using eqn (7). The parameters *t*_maxo_ and *E*_atmax_ were also determined by plotting ln *t*_max_*versus* the reciprocal temperature. The predictions for *t*_max_ were obtained by using eqn (7) and are represented for a long dash line in Fig. 7D. This behavior is similar to the one described in the BR model. The effects of temperature over the entire drug release process of R6G were analyzed using the BR and BRD models of release, and are presented in Fig. 8. In the analysis performed with the BR model given by eqn (14), the parameters *k*_b_, *k*_r_, and *t*_max_ were calculated using eqn (3), eqn (6), and eqn (7), respectively. Fig. 8A describes different possible profiles of drug release in the range of temperatures from 33 °C to 60 °C, when the BR model is used. The effect of temperature in the overall drug release, using the BRD model eqn (15), was also determined, where the parameters *k*_b_, *k*_r_, *t*_max_, and *D*_e_ were calculated using eqn (3), eqn (6), eqn (7), and eqn (13), respectively.

Different profiles of drug release in the range of temperatures from 33 °C to 60 °C, with the BRD model are shown in Fig. 8B. The values of *R*^2^ and the *R*_adjusted_^2^ were used to compare the outcome of the two models. Outstanding fit values for *R*^2^ and *R*_adjusted_^2^ were obtained for both the BR and BRD models and given in Tables 1 and 2, respectively. These results indicate that both models are adequate to describe temperature effects over the release of R6G. The model of BR considers two mechanisms of release: initial burst and nanoparticle degradation, while the BRD model includes a third mechanism of release, diffusion of the drug. Correspondingly, the BR model contains a smaller number of parameters (4), compared to the BRD model (6). As a consequence, the fit obtained (*R*_adjusted_^2^) is slightly better for the BR than for BRD model. However, despite the similar results, the physical phenomena of release for PLGA nanoparticles could be explained better with the inclusion of diffusion in the overall drug release process.

## 4 Conclusions

A mathematical analysis that considers the effect of temperature over the entire drug release process from biodegradable nanoparticles was effectively described. The effects of temperature over the drug release were analyzed using two different models that incorporate two or three mechanisms of release. The first model (BR model) incorporates a mechanism of release of initial burst and a mechanism of release due to nanoparticle degradation. The second model (BRD model), additionally couples a third mechanism of release, diffusion of the drug. R6G loaded PEGylated nanoparticles were successfully produced. The release of R6G from the nanoparticles was investigated at 37 °C, 47 °C, and 57 °C, finding a multiphasic release behavior. An increase in the entire drug release process was observed with the increase of temperature. Both models presented a good fit with the experimental data. The effect of temperature over the controlled release parameters for both models was evaluated with a parametrical analysis. Equations analogous to Arrhenius equation were used to associate the temperature with the controlled release parameters. The parametrical analysis of the two models showed that initial burst constant and the nanoparticle degradation constant increase with increasing temperature. On the other hand, the time to reach the maximum release rate decreased when increasing the temperature for both models. The effective diffusion coefficient presents a direct correlation with temperature for the BRD model. The proposed models are adequate in describing the drug release phenomena and showed good prediction of the experimental data with the temperature range in this study. However, the physical phenomena of drug release will most likely contain a diffusion effect due to changes in the physical properties of encapsulated drugs and their interaction with polymer matrices.

## Conflicts of interest

The authors have no conflicts to declare.

## Supplementary Material
